# A novel mutant p53 binding partner BAG5 stabilizes mutant p53 and promotes mutant p53 GOFs in tumorigenesis

**DOI:** 10.1038/celldisc.2016.39

**Published:** 2016-11-01

**Authors:** Xuetian Yue, Yuhan Zhao, Grace Huang, Jun Li, Junlan Zhu, Zhaohui Feng, Wenwei Hu

**Affiliations:** 1 Department of Radiation Oncology, Rutgers Cancer Institute of New Jersey, Rutgers the State University of New Jersey, New Brunswick, NJ, USA; 2 Department of Environmental Medicine, Nelson Institute of Environmental Medicine, New York University School of Medicine, Tuxedo, NY, USA; 3 Department of Pharmacology, Rutgers the State University of New Jersey, New Brunswick, NJ, USA

**Keywords:** BAG5, metastasis, mutant p53 GOFs, ubiquitination

## Abstract

Tumor suppressor p53 is the most frequently mutated gene in human tumors. Many tumor-associated mutant p53 (mutp53) proteins gain new tumor-promoting activities, including increased proliferation, metastasis and chemoresistance of tumor cells, which are defined as gain-of-functions (GOFs). Mutp53 proteins often accumulate at high levels in human tumors, which is important for mutp53 to exert their GOFs. The mechanism underlying mutp53 proteins accumulation in tumors is not fully understood. Here, we report that BAG5, a member of Bcl-2-associated athanogene (BAG) family proteins, promotes mutp53 accumulation in tumors, which in turn enhances mutp53 GOFs. Mechanistically, BAG5 interacts with mutp53 proteins to protect mutp53 from ubiquitination and degradation by E3 ubiquitin ligases MDM2 and CHIP, which in turn promotes mutp53 protein accumulation and therefore GOFs in promoting cell proliferation, tumor growth, cell migration and chemoresistance. BAG5 is frequently overexpressed in many human tumors and the overexpression of BAG5 is associated with poor prognosis of cancer patients. Altogether, this study revealed that inhibition of mutp53 degradation by BAG5 is a novel and critical mechanism underlying mutp53 protein accumulation and GOFs in cancer. Furthermore, our results also uncovered that promoting mutp53 accumulation and GOFs is a novel mechanism of BAG5 in tumorigenesis.

## Introduction

Tumor suppressor p53 exerts its tumor suppressive function through regulation of a variety of biological processes, including cell cycle arrest, DNA repair, apoptosis, senescence, angiogenesis, metastasis, energy metabolism and response to chemotherapy [1, 2]. In human tumors, p53 is the most frequently mutated gene; ~50% of all human tumors contain mutation in the p53 gene [3, 4]. Most of the p53 mutations occur in its DNA binding domain (DBD). Many tumor-associated mutant p53 (mutp53) proteins not only lose the tumor suppressive function of wild type p53 (wtp53), but also gain new functions in promoting tumorigenesis, which are defined as gain-of-functions (GOFs). Many mutp53 GOFs have been identified, which include the ability to promote cell proliferation, metastasis and chemoresistance [5, 6].

One unique feature of mutp53 is that mutp53 protein levels often accumulate to very high levels in tumor cells/tissues due to increased protein stability [7, 8]. The stabilization and accumulation of mutp53 protein are critical for mutp53 proteins to exert their GOFs in tumorigenesis [7, 8]. The mechanism of increased mutp53 protein stabilization and accumulation in tumors is far from clear. Therefore, it is important to understand the underlying mechanism of mutp53 protein accumulation in tumors, which can help in developing novel therapeutic strategies to target tumors with mutp53, which often have poor therapeutic response and prognosis.

It is worth noting that although mutp53 proteins accumulate at high levels in cancer cells/tissues, they do not possess inherited stability in normal cells/tissues, suggesting that cancer cells provide an environment that promotes the stability and accumulation of mutp53 proteins [7, 8]. MDM2, an E3 ubiquitin ligase, is a key negative regulator of wtp53 protein, which binds to and degrades wtp53 through ubiquitination [9, 10]. Studies including ours have shown that MDM2 retains the ability to negatively regulate mutp53 to keep low mutp53 protein levels in normal tissues [11, 12]. In addition to MDM2, mutp53 can be degraded by E3 ubiquitin ligase CHIP [13]. It is plausible to hypothesize those changes in tumors block mutp53 degradation mediated by MDM2 and CHIP, thereby leading to mutp53 accumulation in tumors. Recently, we found that BAG2 (Bcl-2-associated athanogene 2), a family member of BAG proteins, binds specifically towards mutp53 but not wtp53 through its BAG domain. The BAG2–mutp53 interaction blocks MDM2 from binding to and ubiquitinating mutp53, which in turn promotes mutp53 accumulation and GOF in tumorigenesis [14]. Although findings from this study revealed an important mechanism for mutp53 accumulation in tumors, it raised an interesting question: whether additional proteins containing BAG domain, especially those in the BAG family, have a similar function to interact with mutp53 protein and promote mutp53 accumulation and GOF in tumorigenesis? BAG family contains six family members (BAG1-BAG6), which all contain at least one BAG domain. The BAG family proteins are a multifunctional group of proteins that have diverse functions in many biological processes. BAG domain mediates direct interaction with the ATPase domain of Hsp70/Hsc70 molecular chaperones. In turn, BAG family proteins can function as adapter or co-chaperon proteins forming complexes with proteins in important signaling and have been reported to have a role in neurodegenerative diseases, viral infection and tumorigenesis [15, 16]. However, the precise role and the underlying mechanisms of BAG family proteins in tumorigenesis are poorly understood. In our recent effort to search for mutp53-binding proteins in tumors, we performed an unbiased high throughput screening in tumors from mutp53 R172H knock-in mice by employing immuno-precipitation (IP) with p53 antibody combined with liquid chromatography-tandem mass spectrometry (LC–MS/MS) assays [14]. Results from this screen revealed that in addition to BAG2, BAG5 is also a mutp53-binding partner (Table 1). Among all BAG family members, BAG5 is the only protein that contains more than one BAG domains; BAG5 contains five BAG domains [17, 18]. Currently, the role of BAG5 in tumorigenesis and its underlying mechanisms are poorly understood. It has been reported that BAG5 is overexpressed in human prostate tumor tissues [19]. Overexpression of BAG5 can inhibit ER stress-induced apoptosis in human prostate cancer cells [19]. It has been suggested that BAG5 inhibits apoptosis through the up-regulation of Bcl-2 and Bcl-xl [20]. These results suggest a potential important role of BAG5 in tumorigenesis.

In this study, we found that BAG5 has an important role in promoting mutp53 protein accumulation and GOFs. BAG5 is a specific binding partner of mutp53 protein. BAG5–mutp53 interaction inhibited the ubiquitination and degradation of mutp53 protein mediated by MDM2 and CHIP, which in turn stabilized mutp53 protein and promoted mutp53 GOFs in cell proliferation, tumor growth, metastasis and chemoresistance. BAG5 is frequently overexpressed in many types of human tumors, and BAG5 overexpression is correlated with poor prognosis. Furthermore, we found that BAG5 cooperates with BAG2 to promote mutp53 accumulation and GOFs. Overexpression of both BAG5 and BAG2 exhibits a more significant correlation with poor prognosis compared with BAG5 overexpression or BAG2 overexpression alone. These findings revealed an important mechanism for mutp53 accumulation in tumors and an unidentified and critical role of BAG5 in tumorigenesis.

## Results

### BAG5 preferentially interacts with mutp53 compared with wtp53 protein

**Figure 1:**
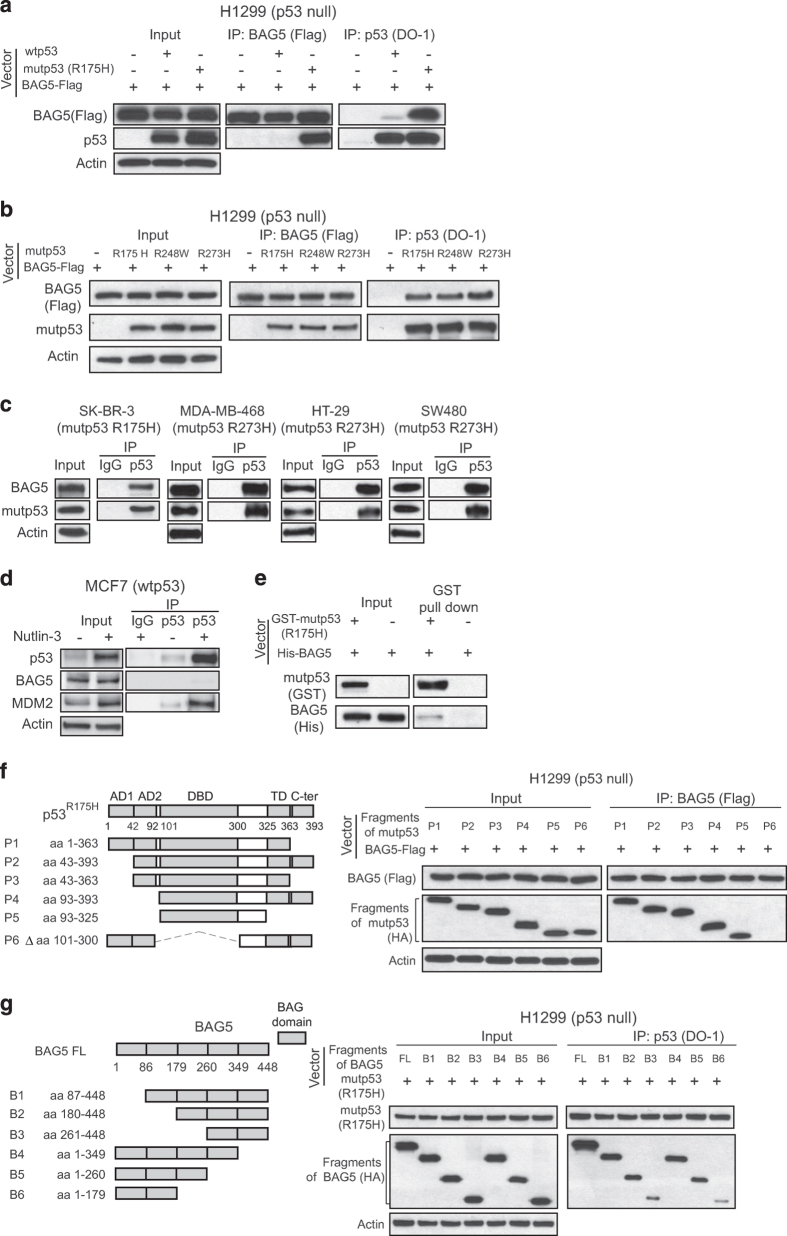
BAG5 is a novel mutp53-binding protein in human cancer cell lines. (**a**) BAG5 preferentially bound to ectopically expressed mutp53 (R175H) compared with wtp53 in human p53-null H1299 cells. H1299 cells were transiently transfected with Flag-tagged BAG5 expression vectors together with wtp53 or mutp53 (R175H) expression vectors. Antibodies used for the IP assays: Flag for Flag-BAG5 and DO-1 for mutp53 and wtp53. (**b**) BAG5 interacted with three hotspot mutp53 proteins, including R175H, R248W and R273H, in H1299 cells. (**c**) Endogenous BAG5 interacted with mutp53 in SK-BR-3 (R175H), MDA-MB-468 (R273H), HT-29 (R273H) and SW480 (R273H) cells as determined by co-IP assays using DO-1 antibody. (**d**) The interaction between BAG5 and wtp53 was barely detectable in MCF7 cells with or without Nutlin 3a (10 μm) treatment as determined by co-IP assays. (**e**) Direct interaction between BAG5 and mutp53 as determined by *in vitro* GST pull-down assay. GST-tagged mutp53 (R175H) immobilized on glutathione beads was incubated with purified His-tagged BAG5. Bound proteins were used for western blot assays. (**f**) BAG5 interacted with mutp53 (R175H) DBD. Left panel: the domain structure of mutp53 R175H was shown in schematic diagram. Right panel: expression vectors of HA-tagged mutp53 R175H fragments were transfected together with Flag-tagged BAG5 expression vectors into H1299 cells. Flag antibody was used for the IP assay. (**g)** BAG domains of BAG5 interacted with mutp53 R175H in H1299 cells. Left panel: the domain fragments of BAG5 were shown in schematic diagram. Right panel: expression vectors of HA-tagged BAG5 fragments were transfected together with mutp53 R175H expression vectors in H1299 cells. DO-1 antibody was used for the IP assay.

In our recent study, using IP combined with LC–MS/MS assays to pull-down mutp53 protein-binding partners in tumors from mutp53 R172H (equivalent to human R175H mutp53) knock-in mice, we found that BAG5 is a mutp53-binding protein (Table 1) [14]. Here, we investigated whether BAG5 interacts with mutp53 protein in human cancer cells. p53-null human lung cancer H1299 cells were transfected with the expression vector of BAG5-Flag together with wtp53 or mutp53 (R175H). Co-IP assays using either anti-p53 or anti-Flag antibodies demonstrated that BAG5 preferentially bound to mutp53 compared with wtp53 (Figure 1a). In addition to R175H, BAG5 also interacted with other tumor-associated mutp53 proteins including R248W and R273H in H1299 cells, respectively (Figure 1b). The endogenous BAG5–mutp53 interaction was also observed in human breast cancer cell lines SK-BR-3, MDA-MB-468, which contain a single copy of p53 gene with R175H and R273H mutation, respectively, and human colorectal cancer cell lines HT-29, SW480, which contain a single copy of p53 gene with R273H mutation (Figure 1c). By contrast, the interaction between BAG5 and wtp53 was barely detectable in human breast cancer cell line MCF7, which contains wtp53 (Figure 1d). Nutlin 3a is a small molecule inhibitor of the interaction of p53 with MDM2, which leads to the non-genotoxic p53 stabilization and accumulation [21]. In MCF7 cells treated with Nutlin 3a, wtp53 protein was accumulated to high levels. However, only a very weak interaction between BAG5 and wtp53 can be observed in MCF7 cells with wtp53 accumulation induced by Nutlin 3a (Figure 1d). To investigate whether BAG5 directly interacts with mutp53, we performed an *in vitro* GST pull-down assay. Using recombinant His-tagged BAG5 and GST-tagged mutp53 (R175H) proteins purified from bacteria, we found that mutp53 (R175H) directly interacted with BAG5 (Figure 1e). It is worth noting that direct BAG5–mutp53 interaction detected in *in vitro* assays is much weaker compared with the BAG5–mutp53 interaction detected *in vivo*, which suggests that the BAG5–mutp53 interaction *in vivo* may involve additional proteins in the complex.

We further fine mapped the regions in BAG5 and mutp53 proteins that are required for their interaction. The expression vectors of full length BAG5 with Flag tag and mutp53 fragments with HA tag were co-transfected into p53-null H1299 cells for IP assays. As shown in Figure 1f, BAG5 interacted with mutp53 fragments which contain DBD, but not the fragments without DBD. The interaction of BAG5 with mutp53 DBD was also observed in DBDs containing different p53 mutations in addition to R175H, including R248W and R273H (Supplementary Figure S1). The region of BAG5 protein required for the BAG5–mutp53 interaction was determined by co-transfection of the HA-tagged BAG5 fragments and full length mutp53 (R175H) expression vectors in H1299 for IP assays. BAG5 has five BAG domains. As shown in Figure 1g, each BAG domain was able to interact with mutp53 and the existence of all BAG domains led to the strongest interaction between mutp53 and BAG5 (Figure 1g). Altogether, these results clearly demonstrated that BAG5 is a binding partner of mutp53, and BAG domains of BAG5 protein and DBD of mutp53 are essential for the BAG5–mutp53 interaction.

### BAG5 increases mutp53 protein levels through inhibiting the ubiquitination and degradation of mutp53 protein mediated by MDM2 and CHIP

**Figure 2:**
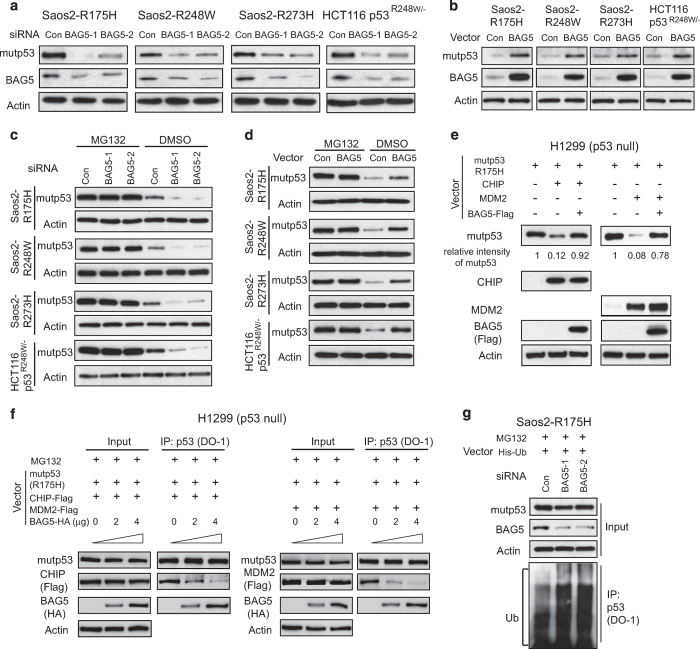
BAG5 inhibits CHIP and MDM2 mediated ubiquitination and degradation of mutp53 to promote mutp53 protein accumulation in human cancer cells. (**a)** Knockdown of endogenous BAG5 by two different siRNA oligos decreased mutp53 protein levels in HCT116p53^*R248W/−*^ and Saos2 cells with stable ectopic expression of mutp53 proteins (R175H, R248W and R273H). The knockdown efficiency of BAG5 at protein level was examined by western blot assays. (**b**) Ectopic expression of Flag-tagged BAG5 increased mutp53 protein levels in cells. (**c**, **d**) MG132 treatment largely abolished the effect of BAG5 on mutp53 protein levels. The effect of BAG5 knockdown on decreasing mutp53 protein levels (**c**) and the effect of ectopic expression of BAG5 on increasing mutp53 protein levels (**d**) were largely abolished in cells treated with MG132 (40 μm) for 6 h. (**e)** BAG5 inhibited CHIP and MDM2 mediated mutp53 (R175H) degradation in H1299 cells. Indicated combination of expression vectors of Flag-tagged BAG5, mutp53 (R175H), MDM2 and CHIP were transfected into H1299 cells. (**f**) BAG5 inhibited the CHIP–mutp53 and MDM2–mutp53 interaction in H1299 cells. Indicated combination of expression vectors of Flag-tagged BAG5, mutp53 (R175H), MDM2 and CHIP were transfected into cells followed by MG132 (40 μm) treatment for 6 h. (**g)** Knockdown of endogenous BAG5 increased the ubiquitination levels of mutp53 in Saos2-R175H cells. Cells were transfected with combination of BAG5 siRNAs and His-Ub expression vectors followed by MG132 (40 μm) treatment for 6 h.

Our recent study found that the BAG2–mutp53 interaction increases mutp53 protein levels through inhibition of mutp53 degradation [14]. Here, we investigated the effect of BAG5 on mutp53 protein level. Endogenous BAG5 was knocked down by two different siRNA oligos in cells with different mutp53 proteins (Saos2-R175H, Saso2-R248W, Saos2-R273H and HCT116*p53^R248W/−^*). The knockdown efficiency of endogenous BAG5 was confirmed at both RNA and protein levels by real-time PCR and western blot assays, respectively (Figure 2a; Supplementary Figure S2). Although BAG5 knockdown had no apparent effect on mutp53 mRNA levels (Supplementary Figure S2), it dramatically decreased mutp53 protein levels (Figure 2a). The effect of BAG5 overexpression on mutp53 protein levels was also examined in the above-mentioned cell lines expressing mutp53. Ectopic BAG5 overexpression clearly increased mutp53 protein levels, but had no apparent effect on mutp53 mRNA levels (Figure 2b; Supplementary Figure S3). These results demonstrated that BAG5 increases mutp53 protein level. To study whether BAG5 increases mutp53 protein level by inhibiting the degradation of mutp53 protein, the above-mentioned cell lines with or without endogenous BAG5 knockdown were treated with MG132, a proteasome inhibitor. Blocking proteasomal degradation by MG132 clearly abolished the effect of BAG5 knockdown on mutp53 protein levels (Figure 2c). Similar results were obtained in cells with ectopic BAG5 overexpression; MG132 treatment abolished the effect of BAG5 overexpression on the mutp53 protein levels (Figure 2d).

MDM2 and CHIP are two major E3 ubiquitin ligases that negatively regulate mutp53 protein levels through proteasomal degradation [12, 13]. We investigated whether BAG5 affects MDM2 and CHIP-mediated mutp53 degradation. Expression vectors of mutp53 (R175H), MDM2, CHIP and BAG5 were employed for transfection in H1299 cells. Ectopic expression of either MDM2 or CHIP substantially decreased mutp53 (R175H) protein levels in H1299 cells. Notably, co-expression of BAG5 largely reduced R175H degradation mediated by MDM2 or CHIP (Figure 2e). We then investigated whether BAG5 inhibits MDM2 and CHIP-mediated mutp53 degradation through inhibiting the mutp53–MDM2 and mutp53–CHIP interaction. The mutp53–MDM2 and mutp53–CHIP interaction was examined in H1299 cells transfected by expression vectors of mutp53 (R175H), MDM2, CHIP along with different amount of BAG5 by co-IP assays. Mutp53 interacted with MDM2 and CHIP. Notably, co-expression of BAG5 clearly decreased the mutp53–MDM2 and mutp53–CHIP interaction in a dose-dependent manner (Figure 2f). These results strongly suggested that BAG5 inhibits MDM2 and CHIP-mediated mutp53 degradation in cells.

To further confirm that BAG5 increases mutp53 protein levels through inhibition of mutp53 ubiquitination and degradation, *in vivo* ubiquitination assay was employed to determine the effect of BAG5 on mutp53 protein ubiquitination levels. Knockdown of endogenous BAG5 by two different siRNA oligos increased the ubiquitination levels of mutp53 R175H in Saos2-R175H cells (Figure 2g). Together, these data demonstrated that BAG5 stabilizes mutp53 protein by inhibiting mutp53 ubiquitination and degradation mediated by MDM2 and CHIP.

### BAG5 promotes mutp53 GOF in tumor growth, metastasis and chemoresistance

**Figure 3:**
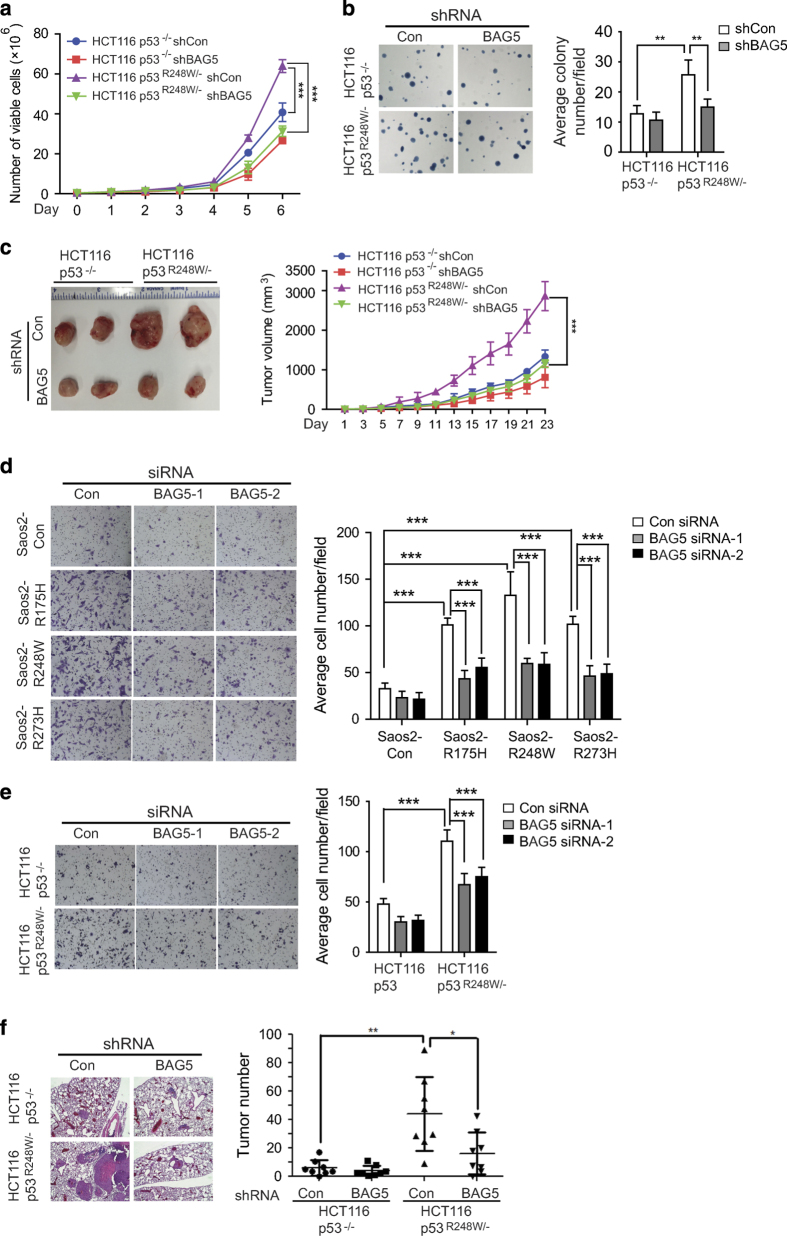
BAG5 promotes mutp53 GOFs in tumor growth and metastasis. (**a**) Knockdown of BAG5 by shRNA greatly inhibited cell proliferation in HCT116*p53*^*R248W/−*^ cells but had a limited effect on HCT116*p53*^*−/−*^ cells. Data are presented as mean±s.d., *n=*3 per group. ***P<0.001; ANOVA followed by student’s *t*-test. (**b**) Knockdown of BAG5 by shRNA clearly inhibited anchorage-independent growth in HCT116*p53*^*R248W/−*^ cells but had a limited effect on HCT116*p53*^*−/−*^ cells. Left panel: representative images of cell colonies in soft agar. Right panel: quantification of average colonies numbers/filed. Data are presented as mean±s.d., *n=*4. **P<0.01; student’s *t*-test. (**c**) Knockdown of BAG5 inhibited xenograft tumor growth in a largely mutp53-dependent manner in HCT116 xenograft tumors. Left panel: representative image of xenograft tumors. Right panel: growth curves of xenograft tumors. Data are presented as mean±s.d., *n=*6 per group. ***P<0.001; ANOVA followed by student’s *t*-test. (**d**) Knockdown of BAG5 by two different siRNA oligos preferentially inhibited the migration ability of Saos2-R175H, Saos2-R248W and Saos2-R273H cells compared with Saos2-con cells as determined by transwell assays. (**e**) Knockdown of endogenous BAG5 preferentially inhibited the migration ability of HCT116*p53*^*R248W/−*^cells compared with HCT116*p53*^*−/−*^ cells. For **d**, **e**, left panels: representative images from a portion of the field; right panels: quantification of average number of migrated cells/field. Data are presented as mean±s.d., *n=*4 per group. ***P<0.001; student’s *t*-test. (**f)** BAG5 knockdown by shRNA greatly inhibited lung metastasis of HCT116*p53*^*R248W/−*^ cells but had a limited effect on HCT116*p53*^*−/−*^ cells *in vivo*. Left panel: representative H&E staining images of lung sections. Right panel: quantification of average number of tumor/lung. Data are presented as mean±s.d., *n=*8 per group. *P<0.05; **P<0.01; student’s *t*-test.

Mutp53 accumulation in tumors is critical for mutp53 GOFs in tumorigenesis [11, 22]. Considering the observation that BAG5 increases mutp53 protein levels, we investigated whether BAG5 promotes mutp53 GOFs. Promoting tumor growth is an important mutp53 GOF [5, 6]. To define the effect of BAG5 on mutp53 GOF in promoting the growth of tumor cells, we employed HCT116*p53* ^*−/−*^ and HCT116*p53* ^*R248W/−*^ cells with or without knockdown of endogenous BAG5 to compare the cell proliferation rate and the anchorage-independent colony formation ability of these cells. We further measured the growth rate of xenograft tumor formed by these cells. Mutp53 promoted cell proliferation and anchorage-independent growth of HCT116 cells; HCT116*p53*^*R248W/−*^ cells displayed a much faster cell proliferation and anchorage-independent growth compared with HCT116*p53*^*−/−*^ cells (Figure 3a and b). Notably, knockdown of BAG5 inhibited cell proliferation and anchorage-independent growth of HCT116*p53*^*R248W/−*^ cells but had a much limited effect in HCT116*p53*^*−/−*^ cells (Figure 3a and b). Similar results were obtained from the xenograft tumorigenesis assays. The growth rate is faster for HCT116*p53*^*R248W/−*^ xenograft tumors compared with HCT116*p53*^*−/−*^ xenograft tumors (Figure 3c). Notably, knockdown of BAG5 by shRNA greatly inhibited the growth of xenograft tumor formed by HCT116*p53*^*R248W/−*^ but had a very limited effect for HCT116p53^*−/−*^ tumors (Figure 3c).

We further examined the effect of BAG5 on mutp53 GOF in promoting cell migration and tumor metastasis using *in vitro* transwell assays and *in vivo* lung metastasis assays, respectively. Consistent with previous reports including ours [11, 14, 23], mutp53 (R175H, R248W and R273H) promoted the migration ability in Saos2 and HCT116 cells (Figure 3d and e; Supplementary Figure S4). Notably, knockdown of BAG5 either by siRNA oligos or shRNA vectors dramatically reduced mutp53 GOF in promoting migration of these cells (Figure 3d and e; Supplementary Figure S4). For *in vivo* lung metastasis assay, HCT116*p53*^*R248W/−*^ and HCT116*p53*^*−/−*^ cells with stable knockdown of BAG5 and control cells were injected into the nude mice *via* the tail vein for lung metastatic tumorigenesis assays. Consistent with previous reports [11, 14], mutp53 promoted the formation of lung metastatic tumors; nude mice injected with HCT116*p53*^*R248W/−*^ cells formed more tumors in the lung compared with mice injected with HCT116*p53*^*−/−*^ cells (Figure 3f). Notably, knockdown of BAG5 largely abolished the effect of mutp53 GOF on metastasis, but showed a limited effect in HCT116*p53*^*−/−*^ cells (Figure 3f).

**Figure 4:**
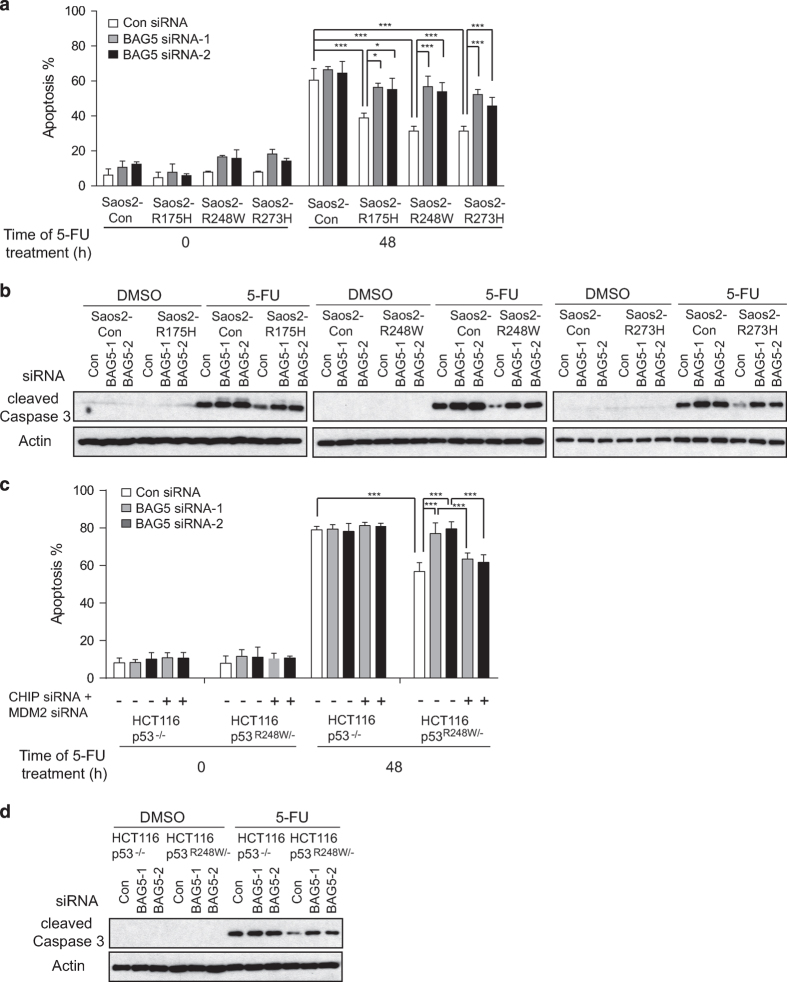
BAG5 promotes mutp53 GOF in enhancing chemoresistance. (**a**, **b**) Knockdown of BAG5 by siRNAs preferentially increased 5-FU induced apoptosis in Saos2 cells with ectopic expression of mutp53 (R175H, R248W and R273H) but had a limited effect in Saos2-con cells. (**a**) The percentage of apoptotic cells was determined by Annexin V assays. Data are present as mean±s.d., *n=*4. *P<0.05; ***P<0.001; student’s *t*-test. (**b**) The degree of apoptosis was examined by the levels of cleaved Caspase 3. (**c**, **d**) Knockdown of BAG5 by siRNAs preferentially increased 5-FU induced apoptosis in HCT116p53^*R248W/−*^ cells but had a limited effect in HCT116*p53*^*−/−*^ cells as determined by Annexin V assays (**c**) and western blot assays for the cleaved Caspase 3 protein levels (**d**). This effect of BAG5 knockdown in HCT116p53^*R248W/−*^ cells was largely abolished when endogenous MDM2 and CHIP were knocked down by siRNA as determined by Annexin V assays (**c**).

One of mutp53 GOFs is to promote chemoresistance [24, 25]. We investigated whether BAG5 regulates mutp53 GOF in chemoresistance. Saos2-R175H, Saos2-R248W, Saos2-R273H, Saos2-con cells and HCT116*p53*^*R248W/−*^, HCT116*p53*^*−/−*^ cells with or without BAG5 knockdown by siRNA were treated with 5-FU, one of the most commonly used chemotherapeutic agent for human cancer. Knockdown of endogenous BAG5 in all these cells did not show significant effect on cell viability (Supplementary Figure S5). Apoptosis induced by 5-FU treatment was measured by Annexin V staining followed by flow cytometry and western blot assays as we described previously [26]. 5-FU induced less apoptosis in mutp53-containing cells lines (Saos2-R175H, Saos2-R248W, Saos2-R273H and HCT116*p53*^*R248W/−*^) compared with their isogenic p53-null control cell lines (Saos2-con and HCT116*p53*^*−/−*^) (Figure 4a–d). Notably, knockdown of BAG5 by two different siRNA oligos greatly increased 5-FU induced apoptosis in cell lines containing mutp53, but had a minimal effect in p53-null cell lines (Figure 4a–d). Further, knockdown of MDM2 and CHIP simultaneously in HCT116*p53*^*R248W/−*^ cells largely abolished the promoting effect of BAG5 knockdown on 5-FU induced apoptosis, which suggests that this effect of BAG5 knockdown is through MDM2 and CHIP-mediated mutp53 degradation (Figure 4c). Altogether, these results clearly demonstrated that BAG5 promotes mutp53 GOFs in tumor growth, metastasis and chemoresistance.

### BAG2 and BAG5 display a cooperative effect on promoting mutp53 protein accumulation and GOF

**Figure 5:**
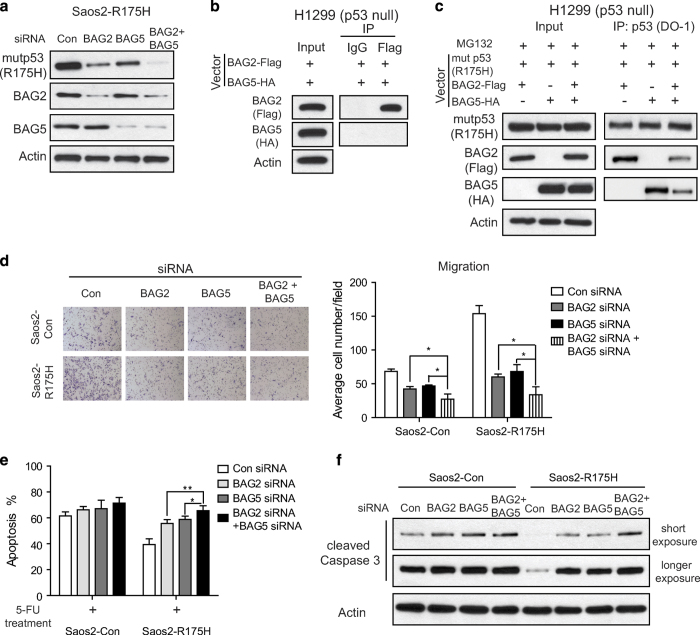
BAG5 and BAG2 cooperate in promoting mutp53 protein accumulation and mutp53 GOFs. (**a**) Simultaneous knockdown of BAG2 and BAG5 by siRNAs reduced mutp53 protein levels to a greater extend compared with individual knockdown of either BAG2 or BAG5 in Saos2-R175H cells. (**b**) No direct protein interaction was observed between BAG2 and BAG5. Expression vectors of Flag-tagged BAG2 and HA-tagged BAG5 were transfected in H1299 cells followed by IP assay. (**c**) BAG2 and BAG5 competed with each other in interacting with mutp53. H1299 cells were transfected with expression vectors of Flag-tagged BAG2 and HA-tagged BAG5 individually or together along with mutp53 (R175H) followed by MG132 (40 μm) treatment for 6 h. (**d**) Simultaneous knockdown of BAG2 and BAG5 exhibited a stronger effect on inhibition of mutp53 GOF in promoting migration than the individual knockdown of either BAG2 or BAG5 in Saos2-R175H cells. Left panels: representative images from a portion of the field; right panels: quantification of average number of migrated cells/field. Data are presented as mean±s.d., *n=*4. *P<0.05; student’s *t*-test. (**e**, **f**) Simultaneous knockdown of BAG2 and BAG5 exhibited a stronger effect on inhibition of mutp53 GOF in chemoresistance than individual knockdown of either BAG2 or BAG5 in Saos2-R175H cells. The levels of 5-FU induced apoptosis were determined by Annexin V assays (**e**) and western blot assays for the cleaved Caspase 3 protein levels (**f**). In **e**, data are presented as mean±s.d., *n=*4. *P<0.05; **P<0.01; student’s *t*-test.

Results from this study and our recent report [14] have shown that both BAG2 and BAG5 can promote mutp53 protein levels and functions. It is unclear whether BAG2 and BAG5 show a cooperative effect on the regulation of mutp53 protein levels and GOFs. To this end, endogenous BAG2 and BAG5 were knocked down in Saos2-R175H cells. Notably, simultaneous knockdown of BAG2 and BAG5 decreased mutp53 protein levels to a greater extent than knockdown of BAG2 or BAG5 individually (Figure 5a). We further studied whether BAG2 and BAG5 interact with mutp53 within a same protein complex. Results from co-IP assays showed that there is no direct interaction between BAG2 and BAG5 in H1299 cells with ectopic expression of BAG2 and BAG5 along with or without mutp53 (R175H) (Figure 5b; Supplementary Figure S6). Interestingly, ectopic BAG2 expression clearly reduced BAG5–mutp53 interaction, and ectopic BAG5 expression also reduced BAG2–mutp53 interaction in H1299 cells, which suggested that BAG2 and BAG5 compete with each other in interacting with mutp53 (Figure 5c).

We further investigated whether BAG2 and BAG5 have a cooperative effect on promoting mutp53 GOFs. Knockdown of endogenous BAG2 and BAG5 simultaneously did not show significant effect on cell viability (Supplementary Figure S7). As shown in Figure 5d, simultaneous knockdown of BAG2 and BAG5 inhibited mutp53 GOF in migration ability to a much greater extend compared with individual knockdown of either BAG2 or BAG5 as determined in Saos2-R175H cells. Similarly, knockdown of BAG2 and BAG5 simultaneously increased 5-FU-induced apoptosis to a much greater extend compared with individual knockdown of either BAG2 or BAG5 in Saos2-R175H cells as determined by flow cytometry and western blot assays (Figure 5e and f). These results demonstrated that BAG2 and BAG5 have a cooperative effect on promoting mutp53 protein accumulation and mutp53 GOF.

### BAG5 overexpression is associated with poor prognosis in cancer patients

**Figure 6:**
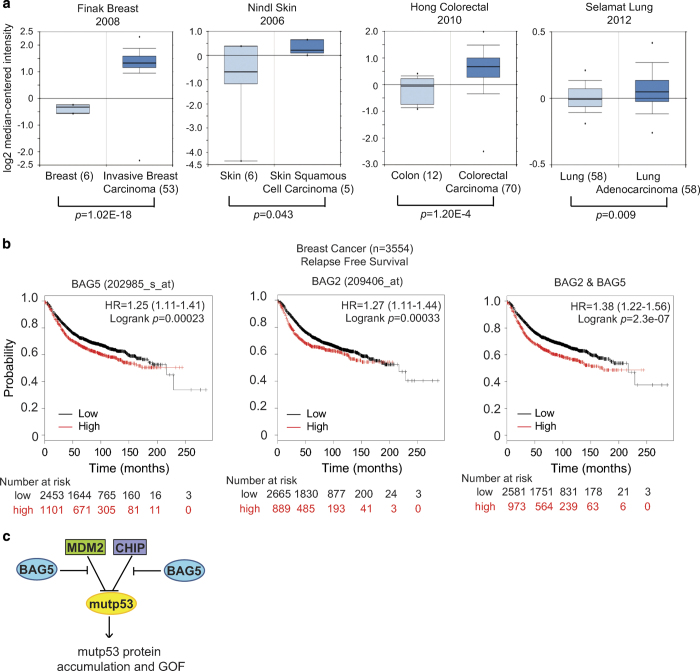
Overexpression of BAG5 is associated with poor prognosis in cancer patients. (**a)** BAG5 mRNA levels are increased in human cancers, including breast cancer, skin cancer, colorectal cancer and lung cancer. The mRNA levels of BAG5 were presented in terms of a log2 median-centered intensity, which was calculated by normalizing the intensity of BAG5 probe to the median of the probe intensities across the entire array. Datasets with BAG5 mRNA levels were obtained from the Oncomine database. (**b**) High levels of BAG5 are associated with poor prognosis in cancer patients. High levels of BAG2 and BAG5 in tumors are associated with a poorer prognosis in cancer patients. Kaplan–Meier curves indicating the relapse free survival of cancer patients separated by the mRNA levels of BAG5 (left panel), BAG2 (middle panel) and both BAG2 and BAG5 (right panel). The survival information and expression levels of BAG2 and BAG5 were obtained from the KM plotter (2014 version for breast cancer). (**c**) A schematic model demonstrating that BAG5 promotes the stabilization and accumulation of mutp53 protein and mutp53 GOFs in tumors through inhibiting the degradation of mutp53 mediated by MDM2 and CHIP.

To elucidate BAG5’s function in tumorigenesis, the expression levels of BAG5 were evaluated in several public available human cancer patient databases. BAG5 is overexpressed in many types of human cancers, including breast cancer, skin cancer, lung cancer and colorectal cancers, compared with normal tissues as analyzed in four databases from Oncomine (Figure 6a). We further analyzed the association of BAG5 expression levels with prognosis in the breast cancer patients by using the KM-plot database. As shown in Figure 6b, high expression levels of BAG5 are associated with poor prognosis in cancer patients. These results strongly suggested that BAG5 promotes tumor progression. Interestingly, the combined prognostic value of BAG2 and BAG5 overexpression is stronger than that of BAG5 overexpression alone (Figure 6b). These results supported the observation that BAG5 cooperates with BAG2 to promote mutp53 accumulation and GOFs, which in turn exhibits a stronger effect on promoting tumorigenesis and is associated with poor prognosis.

## Discussion

p53 is the most frequently mutated gene in human tumors. Ample evidence, including our recent studies, has demonstrated that many tumor-associated mutp53 gain oncogenic function in addition to the loss of tumor suppressive function of wtp53. One characteristic of mutp53 proteins is that mutp53 proteins often stabilize and accumulate at high levels in tumors, which is important for mutp53 to exert GOFs [11, 14]. Therefore, destabilizing mutp53 can greatly reduce mutp53 GOFs in tumorigenesis, which has been actively tested as a very promising therapeutic strategy for tumors containing mutp53 [14, 27–29]. It has been shown that mutp53 protein can be degraded by MDM2 and CHIP, two E3 ubiquitin ligases [11–13]. Studies from transgenic mouse models with knock-in of tumor-associated mutp53 show that mutp53 is kept at low levels in normal tissues and only accumulates in tumor tissues [7, 8, 29]. Currently, the underlying mechanism for mutp53 accumulation is far from clear. Recently, we found that tumor-derived MDM2 short isoforms interfere with MDM2–mutp53 interaction to inhibit MDM2-mediated mutp53 degradation and lead to mutp53 accumulation and enhanced GOF [11]. Our recent study showed that co-chaperone protein BAG2 can also block MDM2–mutp53 interaction, and promote the accumulation of mutp53 protein [14].

In this study, we found that BAG5 has an important role in tumorigenesis by promoting mutp53 protein accumulation and mutp53 GOFs. BAG5 interacts with mutp53 DBD through its BAG domain (Figure 1f and g). The BAG5–mutp53 interaction inhibits the ubiquitination and degradation of mutp53 mediated by both MDM2 and CHIP (Figure 2e–g). We found that overexpression of BAG5 increases mutp53 protein levels, as knockdown of BAG5 decreases mutp53 protein levels in a panel of human tumor cells (Figure 2a and b). Furthermore, BAG5 knockdown reduces mutp53 GOFs in promoting cell proliferation, tumor growth, metastasis and chemoresistance (Figures 3 and 4). BAG5 is frequently overexpressed in many human tumors and overexpression of BAG5 in tumors is associated with poor prognosis (Figure 6a and b). All these results indicated that BAG5 overexpression in tumors promotes the stabilization and accumulation of mutp53 protein and mutp53 GOFs in tumors (Figure 6c). BAG5 has been reported to take part in the development of neurodegenerative diseases, including the Alzheimer’s disease and Parkinson disease, through unclear mechanism [30–32]. However, the biological function of BAG5 in tumors is unclear. A recently study reported that BAG5 is frequently overexpressed in malignant prostate tissues, suggesting the involvement of BAG5 in tumorigenesis [19]. Results from this study demonstrated that BAG5 has an important role in tumorigenesis, and promoting mutp53 accumulation and GOF by BAG5 is an important underlying mechanism.

Both BAG2 and BAG5 belong to the BAG family proteins. A common feature of the BAG family protein is the existence of the BAG domain, which is a functional domain to modulate chaperone activity [33–35]. BAG domain is essential for BAG2–mutp53 interaction and BAG5–mutp53 interaction. We further studied the relationship of BAG2 and BAG5 in regulating mutp53. Although BAG2 and BAG5 do not directly interact with each other (Figure 5b), they show cooperative effect on promoting mutp53 protein accumulation and GOF in tumorigenesis. Simultaneous knockdown of BAG2 and BAG5 in cells reduces mutp53 protein levels and its GOF in promoting migration and chemoresistance to a much greater extent than knockdown of BAG2 and BAG5 individually (Figure 5a and d–f). Data from this study further showed that patients with both BAG2 and BAG5 overexpression have a poorer prognosis than patients with only BAG2 or BAG5 overexpression (Figure 6b). All these data suggested that BAG5 as an oncogene cooperates with BAG2 to promote tumorigenesis.

In addition to BAG2 and BAG5, BAG family protein members include BAG1, BAG3, BAG4 and BAG6. It is unclear why only BAG2 and BAG5 were identified by IP combined with LC–MS/MS assays in searching for mutp53-binding partners in mouse mutp53 tumors. It is worth noting that BAG5 is the only BAG family protein that has five BAG domains, whereas all other BAG family members have only one BAG domain [17, 18]. As to BAG2, it contains a unique BAG domain termed as ‘brand new BAG’ (BNB) domain, which may contribute to the unique binding affinity of BAG2 towards mutp53 [36]. It has been reported that the BAG family proteins have important roles in a wide variety of biological processes, including cell division, cell death and cell differentiation [15, 16]. However, the role of BAG family proteins in tumorigenesis is unclear. It will be interesting to investigate the potential role of other BAG family proteins in tumorigenesis in future study.

In summary, this study identified BAG5 as a novel mutp53-binding protein, which has an important role in mutp53 accumulation and GOFs in tumorigenesis through inhibition of ubiquitination and degradation of mutp53 by MDM2 and CHIP. Results from this study provided a new insight/mechanism for the stabilization and accumulation of mutp53 protein in tumors, and demonstrated the important role of BAG5 in tumorigenesis. The role of BAG5 in promoting mutp53 accumulation and mutp53 GOFs suggests that BAG5 is a potential target for cancer therapy in tumors carrying mutp53.

## Materials and Methods

### Cells, plasmids and cell treatments

Human lung cancer cell line H1299, breast cancer cell lines SK-BR-3, MDA-MB-468, MCF7, colorectal cancer cell lines HT-29, SW480 and osteosarcoma cell line Saos2 were obtained from ATCC. HCT116*p53*^*−/−*^ and HCT116*p53*^*R248W/−*^ were generous gifts from Dr. Bert Vogelstein at Johns Hopkins University. Cell lines with stable ectopic mutp53 (R175H, R248W and R273H) overexpression were established in our lab as described previously [11].

Expression vectors of human MDM2, mutp53 (R175H, R238W and R273H) and HA-tagged mutp53 fragments were generated previously [11, 14, 26, 37]. Flag-tagged BAG5 expression vector was a gift from Dr Lorraine V. Kalia [38]. Flag-tagged BAG5 expression vector was used as a template to construct HA-tagged expression vectors of full length BAG5 and BAG5 fragments (B1- B6) by standard cloning protocol. Flag-tagged CHIP was cloned by the standard cloning protocol. The primers used for cloning are listed in Supplementary Table S1. Lentivirus shRNA vectors against human BAG5 were purchased from Open Biosystems (Thermo Scientific, Waltham, MA, USA, Cat# V2LHS_67687). 5-FU, Nutlin 3a and MG132 were purchased from Sigma (St. Louis, MO, USA).

### IP assays

IP assays were performed as we described previously [11]. In brief, 1×10^6^ p53-null H1299 cells were transfected with different expression vectors. Cells were collected and lysed in NP-40 buffer 24 h after transfection for IP experiments by using anti-p53 antibody (DO-1) (Santa Cruz, Dallas, TX, USA) and anti-Flag (Sigma) antibodies, respectively.

### Western blot assays

Standard western blot assays were used to analyze the levels of protein [14, 37]. Anti-p53 (FL393, 1:2 000 dilution, Santa Cruz), anti-MDM2 (2A10, 1:1 000), anti-Flag (F7425,1:10 000 dilution, Sigma), anti-BAG5(ARP61996-P050, 1:1 000 dilution, Aviva Systems Biology, San Diego, CA, USA), anti-HA (sc-7392, 1:2 000 dilution, Santa Cruz), anti-CHIP (sc-66830, 1:1 000 dilution, Santa Cruz), anti-cleaved-Caspase 3 (D175, 1:1 000 dilution, Cell Signaling, Danvers, MA, USA) and anti-β-actin(A5316, 1:20 000 dilution, Sigma) antibodies were used to determine the levels of p53, MDM2, Flag-BAG5, BAG5, HA-BAG5, CHIP, cleaved caspase 3 and β-actin, respectively.

### Quantitative real-time PCR

Total RNAs of cells were prepared by using RNeasy Mini Kit kit (Qiagen, Valencia, CA, USA). 1 μg RNA was reversely transcribed to cDNA by using a cDNA Reverse Transcription Kit (Applied Biosystems/Thermo Scientific). The expression of genes was detected by Taqman real-time PCR (Applied Biosystems), and normalized with β-actin gene.

### *In vivo* ubiquitination of Mutp53

*In vivo* ubiquitination assays were performed as previously described [14]. In brief, Saos2-R175 cells were transfected with BAG5 siRNAs and His-ubiquitin expression vectors. At 24 h after transfection, cells were treated with MG132 (40 μm) for 6 h. DO-1 antibody (for mutp53) was used to pull-down mutp53, and anti-ubiquitin antibody (P4D1, 1:1 000, Santa Cruz) was used to detect the ubiquitination levels of mutp53 protein.

### Assays for Annexin V staining

Muse Annexin and Dead Cell Assay Kit (Millipore, Billerica, MA, USA) was used to analyze the apoptotic cells [14, 26]. Cells were transfected with control siRNA or siRNA targeting BAG5, then treated with 5-FU for 48 h. Apoptosis was measured by staining cells using Muse Annexin V & Dead Cell Assay Kit (Millipore) and analyzing the cells in a bench flow cytometry, the Muse Cell Analyzer (Millipore), according to manufacturer’s instructions.

### Cell migration assay

The transwell system (BD Biosciences, San Jose, CA, USA) was employed for cell migration assays as described previously [39]. In brief, cells were seeded on the upper chambers in serum-free medium. The lower chambers were filled with the normal culture medium. After culturing for 24 h, cells on the lower surface of upper chambers were fixed by methanol, and stained using crystal violet. The numbers of migration cells was counted in at least five randomly selected fields under an optical microscope by image J software.

### Anchorage-independent growth assays

Anchorage-independent growth assays were performed as previously described [37]. In brief, six-well culture plates were coated with media containing 0.6% agarose on the bottom. Cells (1×10^3^) were seeded with the culture media containing 0.3% agarose. Crystal violet was used to stain the colonies after culturing cells for 10 days.

### Xenograft tumorigenicity assays

The 6-week-old BALB/c nude mice (Taconic) were injected subcutaneously (s.c.) with HCT116p53^*−/−*^ and HCT116p53^*R248W/−*^ cells with or without stable BAG5 knockdown by shRNA vectors. Tumor volume was monitored every 2 days for 3 weeks. The tumor volumes were calculated as follows: volume= (length/2)×width^2^. All animal experiments were approved by IACUC committee of Rutgers University.

### *In vivo* lung metastasis assays

For *in vivo* lung metastasis assays, cells were injected into 8-week-old BLAB/c nude mice *via* the tail vein. Mice were killed at 6 weeks after the inoculation and lungs were removed for subsequent histological assay. The number of lung metastatic tumors was counted under a dissecting microscope and confirmed by histopathological analysis.

### Database of cancer patients

Oncomine database, which has a large collection of publically available databases with gene expression data, was used to analyze the expression level of BAG5 in Breast cancer (GSE9014) [40], skin cancer (GSE2503) [41], colorectal cancer (GSE9348) [42] and lung cancer (GSE32863) patients [43]. The KM plotter online survival database (www.kmplot.com) can assess the effect of gene expression on survival in a large number of breast cancer patients [44]. To analyze the prognostic value of BAG5 and BAG2 expression levels for relapse free survival, the patients collected in 2014 version were split into two groups by using auto-selection of best cutoff method, which uses the best preforming threshold as a cutoff. The Affymetrix IDs used for analysis are: 202985_s_at for BAG5 and 209406_at for BAG2.

### Statistical analysis

The data were present as mean±s.d. The GraphPad Prism 5 software (La Jolla, CA, USA) was used for data analysis. Student’s *t*-test and ANOVA were applied to determine the significant difference two groups. Kaplan–Meier statistics were used to analyze the significant difference of survival of the patients. In all these analyses, *P<0.05* were considered to be significant.

## Figures and Tables

**Table 1:** Candidate mutp53-binding proteins identified in thymic lymphomas from mutant p53 (R172H) mice

	*Gene names*	*Average counts*		*Fold change*	*Reference*
		*Normal thymus*	*Thymic lymphoma*		
Mutp53-binding proteins reported previously	*Hsp70*	1	66	66	PMID:9235949
	*Myosin*	0	8		PMID:24487586
	*Hsp90*	2	15.5	7.7	PMID:15613472
	*Pontin*	4	11	2.75	PMID:25857266
	*Cct8*	36	45	1.25	PMID:23747015
BAG family proteins bound to mutp53	*BAG2*	0	15		
	*BAG5*	7	31.5	4.5	
