# Supplementary material for: A novel mutant p53 binding partner BAG5 stabilizes mutant p53 and promotes mutant p53 GOFs in tumorigenesis
Source: Cell Discov. 2016 Nov 1;2:16039–. doi: 10.1038/celldisc.2016.39 (PMC5088412; doi:10.1038/celldisc.2016.39)

Supplementary materials

Figure Legend for Supplementary Data

Supplementary Figure 1. BAG5 interacted with the DBD of mutp53 proteins (R175H, R248W and R273H). Expression vectors of Flag-tagged BAG5 were transfected together with expression vectors of DBD fragments containing wtp53 or different mutp53 (R175H, R248W and R273H) into H1299 cells. Flag antibody was used for the IP assay.

Supplementary Figure 2. BAG5 knockdown has no apparent effect on mutp53 mRNA levels in human cancer cells. Left panel: the knockdown efficiency of BAG5 by two different siRNAs in Saos2-R175H, Saos2-R248W, Saos2-R273H and HCT116 $p53^{R248W/-}$ cells. Right panel: the mutp53 mRNA levels in cells with and without BAG5 knockdown.

Supplementary Figure 3. Ectopic expression of BAG5 has no apparent effect on mutp53 mRNA levels in human cancer cells. The mRNA levels of BAG5 (left panel) and mutp53 (right panel) in cells with and without ectopic expression of BAG5 were determined by real-time PCR and normalized with the expression levels of β-actin.

Supplementary Figure 4. BAG5 knockdown by shRNA preferentially inhibited the migration ability of HCT116 $p53^{R248W/-}$ cells compared with HCT116 $p53^{-/-}$ cells as determined by transwell assays. Left panel: representative images from a portion of the field; right panel: quantification of average number of migrated cells/field. Data are presented as mean±SD, n=4. **: $p<0.01$; ***: $p<0.001$.

Supplementary Figure 5. BAG5 knockdown by siRNA did not have significant effect on cell viability. Endogenous BAG5 was knocked down by 2 different siRNA in Saos2 cells with ectopic expression of mutp53 and Saos2-control cells (left panel) as well as HCT116 $p53^{R248W/-}$ and HCT116$p53^{-/-}$ cells (right panel). Cell viability was determined by trypan blue exclusion assays using the Vi-CELL cell counter. Data are presented as mean±SD, n=4.

Supplementary Figure 6. No direct protein interaction was observed between BAG2 and BAG5 in H1299 cells with ectopic expression of mutp53 (R175H). Expression vectors of Flag-tagged BAG2, HA-tagged BAG5 and mutp53 (R175H) were transfected in H1299 cells followed by IP assay.

Supplementary Figure 7. BAG2 and BAG5 knockdown by siRNA individually or simultaneously did not have significant effect on cell viability. Cell viability was determined in Saos2-control and Saos2-R175H cells transfected with siRNA targeting BAG2 and BAG5 individually or simultaneously. Data are presented as mean±SD, n=4.

Supplementary Table 1. The oligos used for cloning and sequences of siRNA oligos.

Oloigos name		Oligos sequences
**HA-BAG5**	**Forward**	5'-CGG AAT TCA CCA TGG GCT ACC CAT ACG ATG TTC CAG ATT ACG CTG ATA TGG GAA ACC AAC ATC -3'
	**Reverse**	5'-CGG AAT TCT CAG TAC TCC CAT TCA TCA-3'
**B1, B2 and B3**	**B1 Forward**	5'- CGG AAT TCA CCA TGG GCT ACC CAT ACG ATG TTC CAG ATT ACG CTA ACC ACC CAC ACC GGA TTG A -3'
	**B2 Forward**	5'- CGG AAT TCA CCA TGG GCT ACC CAT ACG ATG TTC CAG ATT ACG CTC ATC CTT CCG TTG CCA AAA -3'
	**B3 Forward**	5'- CGG AAT TCA CCA TGG GCT ACC CAT ACG ATG TTC CAG ATT ACG CTG AAG CAG ACA CAA CTA AA -3'
	**Reverse**	5'-CGG AAT TCT CAG TAC TCC CAT TCA TCA-3'
**B4, B5 and B6**	**Forward**	5'-CGG AAT TCA CCA TGG GCT ACC CAT ACG ATG TTC CAG ATT ACG CTG ATA TGG GAA ACC AAC ATC -3'
	**B4 Reverse**	5'-CGG AAT TCC TAC TTC AAG TCA ATA TAT GT-3'
	**B5 Reverse**	5'-CGG AAT TCC TAC TCT TCC AAA TCC AGA T-3'
	**B6 Reverse**	5'-CGG AAT TCC TAT GCA TCC TCG GAA AGC G-3'
**CHIP-Flag**	**Forward**	5'-CCCAAGCTTACCATGAAGGGCAAGGAGGAGAA-3'
	**Reverse**	5'-CGGGATCCGTAGTCCACCCAGCCATT-3'
**BAG5 siRNA-1**		5'-GCAUUAAAGAGAAGGGAUAAAGGAC-3'
**BAG5 siRNA-2**		5'-GGAGCACCCAUCCCAUAAAGCCGTC-3'
**CHIP siRNA**		5'-AGAAGCGCUGGAACAGCAUUGAGGA-3'

Supplementary Figure 1

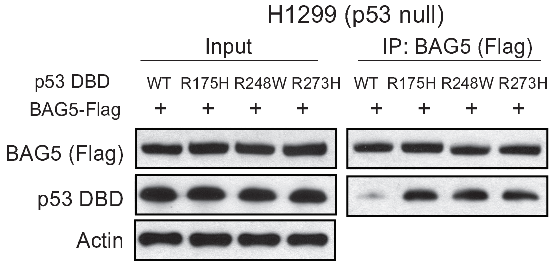

Supplementary Figure 2

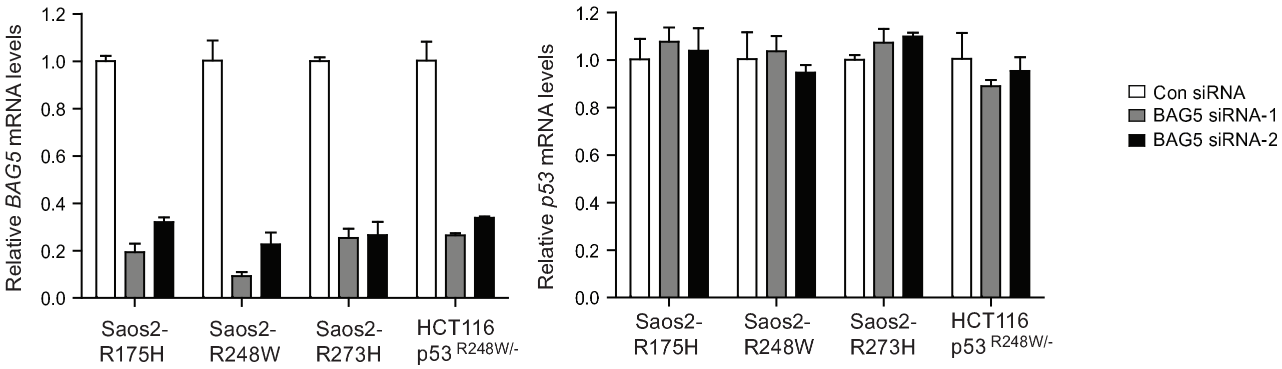

Supplementary Figure 3

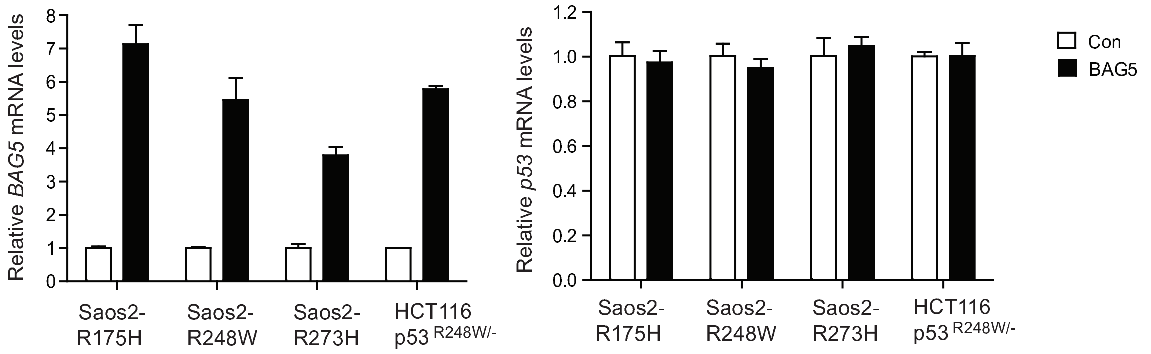

Supplementary Figure 4

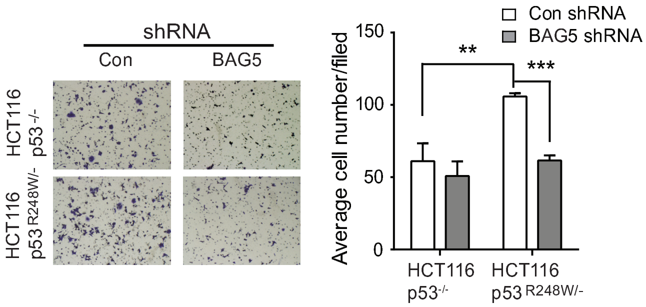

Supplementary Figure 5

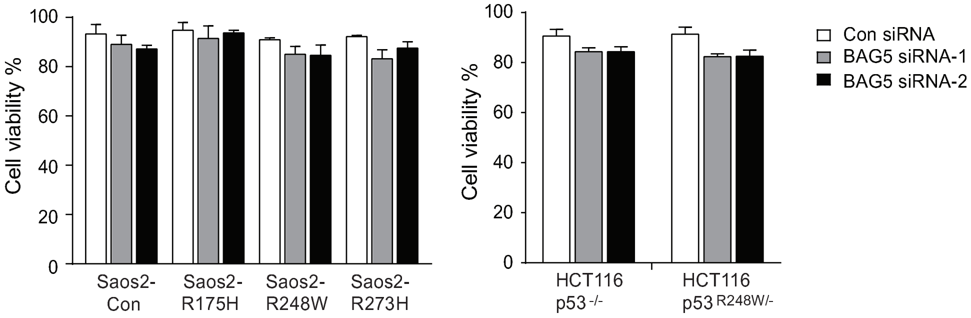

Supplementary Figure 6

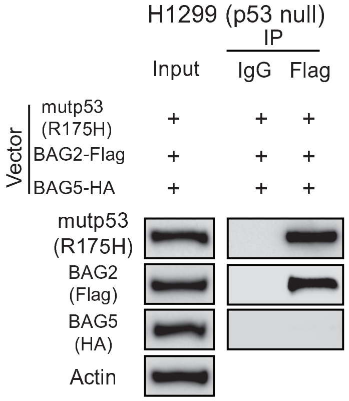

Supplementary Figure 7

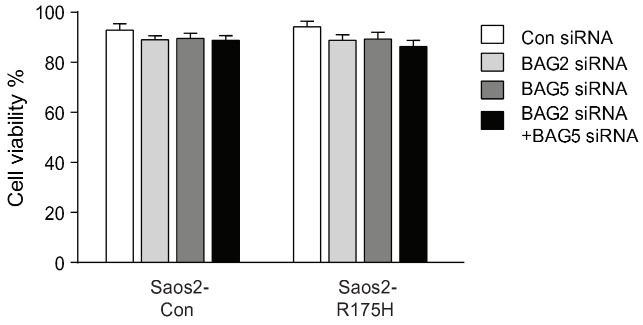

Supplement: Supplementary Information [file celldisc201639-s1.pdf]
